# Distribution and Genetic Profiles of *Campylobacter* in Commercial Broiler Production from Breeder to Slaughter in Thailand

**DOI:** 10.1371/journal.pone.0149585

**Published:** 2016-02-17

**Authors:** Sakaoporn Prachantasena, Petcharatt Charununtakorn, Suthida Muangnoicharoen, Luck Hankla, Natthaporn Techawal, Prapansak Chaveerach, Pravate Tuitemwong, Nipa Chokesajjawatee, Nicola Williams, Tom Humphrey, Taradon Luangtongkum

**Affiliations:** 1 Department of Veterinary Public Health, Faculty of Veterinary Science, Chulalongkorn University, Bangkok, Thailand; 2 Research Unit in Microbial Food Safety and Antimicrobial Resistance, Faculty of Veterinary Science, Chulalongkorn University, Bangkok, Thailand; 3 Department of Veterinary Public Health, Faculty of Veterinary Medicine, Khon Kaen University, Khon Kaen, Thailand; 4 Department of Microbiology, Faculty of Science, King Mongkut’s University of Technology Thonburi, Bangkok, Thailand; 5 National Center for Genetic Engineering and Biotechnology, National Science and Technology Development Agency, Pathumthani, Thailand; 6 Institute of Infection and Global Health, University of Liverpool, Liverpool, United Kingdom; 7 College of Medicine, Swansea University, Singleton Park, Swansea, United Kingdom; USDA-ARS-ERRC, UNITED STATES

## Abstract

Poultry and poultry products are commonly considered as the major vehicle of *Campylobacter* infection in humans worldwide. To reduce the number of human cases, the epidemiology of *Campylobacter* in poultry must be better understood. Therefore, the objective of the present study was to determine the distribution and genetic relatedness of *Campylobacter* in the Thai chicken production industry. During June to October 2012, entire broiler production processes (i.e., breeder flock, hatchery, broiler farm and slaughterhouse) of five broiler production chains were investigated chronologically. Representative isolates of *C*. *jejuni* from each production stage were characterized by *flaA* SVR sequencing and multilocus sequence typing (MLST). Amongst 311 selected isolates, 29 *flaA* SVR alleles and 17 sequence types (STs) were identified. The common clonal complexes (CCs) found in this study were CC-45, CC-353, CC-354 and CC-574. *C*. *jejuni* isolated from breeders were distantly related to those isolated from broilers and chicken carcasses, while *C*. *jejuni* isolates from the slaughterhouse environment and meat products were similar to those isolated from broiler flocks. Genotypic identification of *C*. *jejuni* in slaughterhouses indicated that broilers were the main source of *Campylobacter* contamination of chicken meat during processing. To effectively reduce *Campylobacter* in poultry meat products, control and prevention strategies should be aimed at both farm and slaughterhouse levels.

## Introduction

Campylobacteriosis is one of the most prevalent bacterial gastrointestinal diseases in humans worldwide, particularly in developed countries [1, 2]. *Campylobacter jejuni* is the most frequent species associated with human infection, followed by *C*. *coli*. Although most patients recover spontaneously, serious post-infectious complications, such as Guillain-Barré syndrome, Reactive arthritis and Reiter’s syndrome are sometimes seen [3]. Foods of animal origin, especially poultry and poultry products, are considered as the important sources of human infection [4].

Handling, consumption and preparation of poultry meat are widely referred as the risk factors associated with *Campylobacter* infection in humans. According to the Scientific Opinion of the Panel on Biological Hazards [5], the chicken reservoir was estimated to be responsible for 50–80 percent of human cases. To demonstrate the relationship between *Campylobacter* isolated from humans and poultry, epidemiological investigations using molecular techniques were conducted [6–8]. From a global database, the major clonal complexes of multilocus sequence typing (MLST) identified in campylobacteriosis cases were similar to the main clonal complexes isolated from chickens [9]. Control of *Campylobacter* at a significant source, like poultry meat, is the most effective strategy to reduce the prevalence of *Campylobacter* infection in humans.

Epidemiological studies of *Campylobacter* in poultry have been undertaken worldwide. A high prevalence of *Campylobacter* was found in breeder flocks, however, there was low evidence of these bacteria reported in fertile eggs [10]. In addition, *Campylobacter* isolated from breeders and their progenies were found not to be genetically related [11]. For instance, the study of O’Mahony et al. (2011) [7] identified *flaA* SVR types 15, 16, 18, 22, 34, 57, 66, 239, 964, 1134 and 1136 in breeder flocks but no identical genotype was found in broiler flocks of the same production chain. Similar findings were also reported by other investigations [11, 12]. Therefore, several studies suggested that vertical transmission is not the major route of *Campylobacter* transmission into broiler flocks. In contrast, horizontal transmission is believed to be the important route for *Campylobacter* colonization in broiler flocks [13]. The presence of potential sources of *Campylobacter* in the broiler house environment was reported as the significant risk factor for *Campylobacter* colonization in broilers [12]. Farm staff and pests can act as vehicles for ingress of *Campylobacter* into broiler flocks [14]. In addition, water supplied to the birds, particularly untreated water, was reported as a source of *Campylobacter* on broiler farms [15]. Similarity of sequence types identified in broilers and puddles in the study of Bull et al. (2006) emphasized the possibility of the environment surrounding the house as one of the potential sources of *Campylobacter*. During transportation, transport vehicles and crates can also represent important routes of *Campylobacter* into the poultry production process [16]. Frequently, *Campylobacter* contaminating slaughterhouse equipment and meat products were genetically identical to those of broiler flocks [17]. Visceral rupture and insufficient disinfection of equipment have been found to be the major sources of *Campylobacter* contamination in the slaughterhouse [18, 19].

To reduce the contamination of *Campylobacter* in poultry meat products, routes of *Campylobacter* transmission during the broiler production process should be clarified. To date, no longitudinal investigation of *Campylobacter* in Thai broiler flocks has been undertaken. Therefore, the objective of this study was to determine the distribution and genetic relatedness of *Campylobacter* in the Thai chicken production industry. Five integrated broiler production chains in Thailand were examined longitudinally from breeder farm to slaughterhouse. *flaA* short variable region (*flaA* SVR) sequencing and multilocus sequence typing (MLST) were used to characterize the genotypes of *C*. *jejuni* isolates in the present study.

## Materials and Methods

### Ethics statement

The sample collection protocol of this study was approved by Chulalongkorn University Animal Care and Use Committee (CU-ACUC). However, the animal use permission number was not available for this study because the committee considered that no severely invasive experiment was applied to animals (official document no. 77/2556). Cloacal swab sampling was performed by well-trained veterinarians according to the recommendations of the CU-ACUC. Discussion between researchers, company staff and farmers was organized prior to the beginning of sampling. The sampling permission was verbally obtained from all companies and farmers participating in this study.

### Farms description

During June to October 2012, five chicken production chains (i.e., A, B, C, D and E), which belonged to two integrated poultry production companies, were chronologically investigated from breeder farm to slaughterhouse. Breeder farms were located distantly from their progeny farms even when affiliated with the same company. Fertile eggs from breeder farms A, B and C were sent to the same hatchery, while breeder farms D and E supplied eggs to another hatchery. Broiler farms A, D and E were located in the eastern region of Thailand, whereas broiler farms B and C were located in the central region and are located next to each other. The size of the broiler farms ranged from 11,200 square meters (farm D) to 32,000 square meters (farm B). Broiler farm A was an antibiotic-free farm with 2 houses and a production capacity of 100,000 chickens per year. Farm B consisted of 10 houses and produced approximately 1,000,000 chickens per year, while farm C was composed of 7 houses and produced around 700,000 chickens per year. Unlike farms A, B and C, broiler farms D and E had only 1 house with a production capacity of 93,000 and 60,000 chickens per year, respectively. Among participating broiler farms, broiler farm A had the lowest number of chickens per house (9,792 birds). The highest number of chickens per house was reported in broiler farm C (26,520 birds per house), followed by broiler farms B (24,480 birds per house), D (16,320 birds per house) and E (11,220 birds per house). Slaughter age of studied broiler flocks ranged from 32 to 42 days. Farms A, B and C were slaughtered in large scale processing plants, whereas farms D and E were slaughtered in a small scale plant.

Chicken production units in this study were located in 5 provinces of Thailand i.e., Nakhon Ratchasima, Lopburi, Prachinburi, Chachoengsao and Samut Sakhon. Breeder flocks were located in 2 provinces, including Phatthana Nikhom district, Lopburi province (14.905180, 101.025927) and Sikhio district, Nakorn Ratchasima province (14.973411, 101.592127). For two hatcheries participating in the present study, one hatchery was located in Phatthana Nikhom district, Lopburi province (14.996333, 100.988580) and the other hatchery was located in Pak Thong Chai district, Nakhon Ratchasima province (14.671060, 102.026135). Broiler farms A, D and E were located in different districts of Prachinburi province i.e., Na Di district (14.203045, 101.775171); Prachantakham district (14.064487, 101.515607) and Kabin Buri district (13.905011, 101.797961), respectively. Both broiler farms B and C were located in Nong Muang district, Lopburi province (15.393529, 100.661319 for farm B and 15.390984, 100.659260 for farm C). Three slaughterhouses were located in Om Noi district, Samut Sakhon province (13.547522, 100.274396); Phatthana Nikhom district, Lopburi province (14.795521, 100.912899) and Phanom Sarakham district, Chachoengsao province (13.744013, 101.346990).

### Sample collection

**Fig 1:**
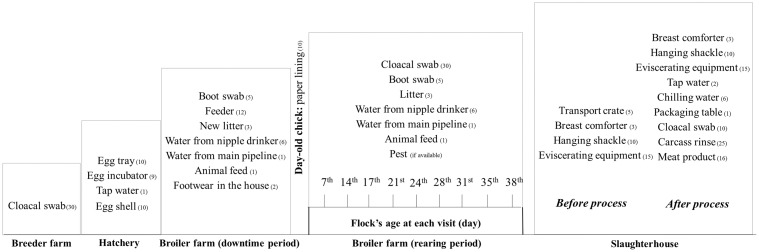
Types and number of samples collected throughout the chicken meat production chain. Area of boot swab sampling at downtime period included anteroom of the target house, inside the target house and area around the house. Area of boot swab sampling during the rearing period included path-leading to the house, anteroom of the target house, inside the target house, area around the house and inside the adjacent house. Flocks D and E were visited at 7^th^, 14^th^, 21^st^, 28^th^, 35^th^ day of the rearing period, while other flocks were visited at 7^th^, 14^th^, 17^th^, 21^st^, 24^th^, 28^th^, 31^st^, 35^th^, 38^th^ day of the rearing period.

In this study, samples were collected from breeder farm to slaughterhouse. Approximately, 2,475 samples from breeder flocks, hatcheries, broiler flocks and slaughterhouses were collected from five production chains (Table 1 and Fig 1). *Campylobacter* colonization in breeder flocks was determined by testing cloacal swab samples. Eggs produced from previously sampled breeder flocks were tracked to hatcheries. Egg trays and egg incubators exposed to target egg batches were swabbed on their surface. Egg shells were randomly taken after chicks were hatched. Prior to chick placement, environmental samples of disinfected houses were collected to determine that they were free of contamination with *Campylobacter* (Fig 1). Faeces-soiled tray liners were collected on the day of chick arrival at the broiler farms and broiler flocks were visited regularly during the rearing period as described in Fig 1. Cloacal swabs from live birds and environmental samples (litter, water from nipple drinkers, water inlet and shoe covers) were taken on each visit. Insects and other pests in farming area were captured as available.

**Table 1:** Number of samples collected throughout chicken production process of 5 production chains.

	Number of samples collected from production chain				
	A	B	C	D	E
**Breeder flock**					
- Cloacal swabs	30	30	30	24	24
**Hatchery**					
- Equipment and environmental samples (e.g., egg tray, incubator and tap water)	27	27	27	27	23
**Broiler house**					
***Before rearing period***					
- Boot swab samples^a^	3	4	4	4	4
- House equipment and environmental samples (e.g., feeder, litter, boots and water)	25	25	25	25	24
***During rearing period***					
- Tray liners	10	10	10	10	10
- Boot swab samples^b^	29	34	34	30	30
- House equipment and environmental samples (e.g., litter, water, pests and feed)	56	50	60	76	75
- Cloacal swabs	210	210	240	150	150
**Slaughterhouse**					
- Cloacal swabs	5	15	15	5	5
- Equipment and environmental samples (e.g., shackle, chilling water, tap water, etc.)^c^	121	122	122	72	97
**Total**	516	527	567	423	442

^a^ Area of boot swab sampling at downtime period: anteroom of the target house, inside the target house and area around the house.

^b^ Area of boot swab sampling during the rearing period: path-leading to the house, anteroom of the target house, inside the target house, area around the house and inside the adjacent house.

^c^ Samples were collected before and during slaughtering process of the selected flock.

Disinfected transport crates before being used were swabbed at the slaughterhouse. Slaughterhouse equipment was sampled before and after the target flock was processed (Fig 1). Three areas on breast comforters were randomly swabbed lengthwise. Shackles were sampled at the hanging and evisceration areas. Evisceration equipment and packaging tables were swabbed thoroughly. Water samples were collected from the bird washing machine, inside/outside washing machine and chiller tanks. For chicken related samples, cloacal swabs from live birds were collected before they were slaughtered. Carcass rinses were performed after scalding, plucking, evisceration, inside/outside washer and chilling steps using buffered peptone water. Intact caeca were randomly taken at the evisceration area. Meat products from post-chilled chicken i.e., breasts, thighs, wings and fillets were collected. All samples were kept on ice and processed within 4 h after sampling.

### *Campylobacter* isolation and identification

Samples were examined by direct plating and selective enrichment methods. The direct plating method was used for *Campylobacter* isolation from cloacal swabs and caecal samples [20]. In brief, samples were streaked directly onto *Campylobacter* blood-free selective agar or mCCDA (CM0739; Oxoid Ltd., Basingstoke, Hampshire, United Kingdom) supplemented with *Campylobacter* selective supplement (cefoperazone, 32 mg/litre and amphotericin B, 10 mg/litre). Samples were incubated at 42°C for 48 h under microaerobic conditions (5% O_2_, 10% CO_2_ and 85% N_2_).

Environmental and meat samples were examined by a selective enrichment culture method [21]. Samples were transferred to Exeter broth consisting of nutrient broth No. 2 (CM0067; Oxoid Ltd., Basingstoke, Hampshire, United Kingdom), *Campylobacter* growth supplement (sodium metabisulphite, 250 mg/litre; sodium pyruvate, 250 mg/litre and ferrous sulfate, 250 mg/litre), *Campylobacter* selective supplement (trimethoprim, 10 mg/litre; rifampicin, 5 mg/litre; polymyxin B, 2,500 IU/litre; cefoperazone, 15 mg/litre and amphotericin B, 2 mg/litre) and 5% sheep blood. One part of animal feed, egg shell, litter, meat products and chilling water were put into nine parts of Exeter broth. Cotton swabs were immersed into 10 ml of broth. A litre of clean water samples (drinking water and tap water) were filtered through 0.45 μm membrane filters (GN-6 Metricel^®^, Pall, USA), and then the membrane filters were immersed in the 20 ml of Exeter broth. Pooled insect samples (darkling beetles and house flies) were crushed, and then added in 10 ml of broth [8]. Rodents and lizards were tested for *Campylobacter* in their faeces and on their skin surface, respectively [22]. Enrichment broths inoculated with samples were incubated under microaerobic conditions for 48 h at 37°C [21]. Thereafter, enriched samples were spread onto mCCDA and incubated at 42°C for 48 h under microaerobic conditions. Presumptive *Campylobacter* colonies were confirmed by multiplex polymerase chain reaction according to previous publications [23–25]. Confirmed isolates were stored in skimmed milk with 30% glycerol at -80°C for further study.

### Genetic characterization

Colonies of *C*. *jejuni* isolated from each production unit were primarily subtyped by *flaA* short variable region. Representatives of *flaA* SVR genotypes were further characterized by multilocus sequence typing (MLST). DNA extraction procedure was performed using Wizard^®^ Genomic DNA purification kit (Promega, Madison, USA).

Short variable region of *flaA* gene was amplified with primers FLA242FU (5’-CTA TGG ATG AGC AAT TWA AAA T-3’) and FLA625RU (5’-CAA GWC CTG TTC CWA CTG AAG-3’) as previously described [26]. PCR products were purified by NucleoSpin^®^ Gel and PCR Clean-up kit (MACHEREY-NAGEL, Düren, Germany) and sent for DNA sequencing at First BASE Laboratories (Selangor Darul Ehsan, Malaysia). To determine allelic numbers, nucleotide sequences were submitted into the online database (http://pubmlst.org/campylobacter/flaA/) [27].

MLST was performed according to the previously published protocol [28]. Internal fragments of seven housekeeping genes (i.e., *aspA*, aspartase A; *glnA*, glutamine synthetase; *gltA*, citrate synthase; *glyA*, serine hydroxymethyltransferase; *pgm*, phosphoglucomutase; *tkt*, transketolase; and *uncA*, ATP synthase α subunit) were amplified and sequenced. Allele numbers, sequence types (STs) and clonal complexes (CCs) were assigned according to the *Campylobacter* MLST database [27]. Phylogenetic reconstruction using the neighbour joining method was performed by importing trimmed sequences into Molecular Evolutionary Genetics Analysis (MEGA) software version 5.2.1 [29].

### Statistical analysis

Prevalence of *Campylobacter* in the same production unit among 5 broiler production chains (i.e., A, B, C, D and E) was compared using chi-square test. Statistical significance was considered at *p*≤0.05.

## Results

### Distribution of *Campylobacter* in Thai poultry production chain

Out of 2,475 examined samples, 608 samples were positive for *Campylobacter* species (24.57%). Overall, the prevalence in breeders, broiler farms and slaughterhouses were 64.49, 16.41 and 43.52%, respectively. In breeder flocks, the proportion of flocks colonized with *Campylobacter* ranged from 36.67 to 76.67% (Table 2). The prevalence of *Campylobacter* in breeder farm A was significantly lower than that in breeder farms B, C, D and E. Isolates obtained from breeder flocks were mainly identified as *C*. *coli*. No *Campylobacter* was detected in hatchery-related samples i.e., egg incubators, egg trays, tap water and egg shell. Likewise, *Campylobacter* were absent in faeces-soiled lining papers and environmental samples from the broiler house before chick placement.

**Table 2:** Distribution of *Campylobacter* in 5 chicken meat production chains in Thailand.

Production chain	Production unit	Chicken-related sample^1^			Environmental sample^2^		
		No. of positive samples /Total (%)	Species identification (%)		No. of positive samples /Total (%)	Species identification (%)	
			*C*. *jejuni*	*C*. *coli*		*C*. *jejuni*	*C*. *coli*
A	Breeder farm	11/30 (36.67)^a^	5/11 (45.45)^a^	6/11 (54.55)^a^	NS^3^	NS	NS
	Hatchery	0/10 (0.00)	n/a^4^	n/a	0/17 (0.00)	n/a	n/a
	Broiler farm	58/220 (26.36)^a^	58/58 (100.00)^5^	0/58 (0.00)	7/113 (6.19)^b^	7/7 (100.00)	0/7 (0.00)
	Slaughterhouse	45/56 (80.36)^a^	45/45 (100.00)	0/45 (0.00)	13/70 (18.57)^b^	13/13 (100.00)	0/13 (0.00)
B	Breeder farm	23/30 (76.67)^b^	6/23 (26.09)^a^^b^	17/23 (73.91)^a^^b^	NS	NS	NS
	Hatchery	0/10 (0.00)	n/a	n/a	0/17 (0.00)	n/a	n/a
	Broiler farm	80/220 (36.36)^b^	80/80 (100.00)	0/80 (0.00)	0/113 (0.00)^a^	n/a	n/a
	Slaughterhouse	34/66 (51.52)^b^	34/34 (100.00)	0/34 (0.00)	27/71 (38.03)^a^	27/27 (100.00)	0/27 (0.00)
C	Breeder farm	21/30 (70.00)^b^	8/21 (38.10)^a^^b^	13/21 (61.90)^a^^b^	NS	NS	NS
	Hatchery	0/10 (0.00)	n/a	n/a	0/17 (0.00)	n/a	n/a
	Broiler farm	2/250 (0.80)^c^	2/2 (100.00)	0/2 (0.00)	1/123 (0.81)^a^^b^	1/1 (100.00)	0/1 (0.00)
	Slaughterhouse	25/66 (37.88)^b^	25/25 (100.00)	0/25 (0.00)	25/71 (35.21)^a^	25/25 (100.00)	0/25 (0.00)
D	Breeder farm	17/24 (70.83)^b^	2/17 (11.76)^b^	15/17 (88.24)^b^	NS	NS	NS
	Hatchery	0/10 (0.00)	n/a	n/a	0/17 (0.00)	n/a	n/a
	Broiler farm	32/160 (20.00)^a^	32/32 (100.00)	0/32 (0.00)	4/135 (2.96)^b^	4/4 (100.00)	0/4 (0.00)
	Slaughterhouse	36/40 (90.00)^a^	36/36 (100.00)	0/36 (0.00)	11/37 (29.73)^a^^b^	11/11 (100.00)	0/11 (0.00)
E	Breeder farm	17/24 (70.83)^b^	8/17 (47.06)^a^	9/17 (52.94)^a^	NS	NS	NS
	Hatchery	0/6 (0.00)	n/a	n/a	0/17 (0.00)	n/a	n/a
	Broiler farm	78/160 (48.75)^d^	78/78 (100.00)	0/78 (0.00)	5/133 (3.76)^b^	5/5 (100.00)	0/5 (0.00)
	Slaughterhouse	32/40 (80.00)^a^	32/32 (100.00)	0/32 (0.00)	4/62 (6.45)^c^	4/4 (100.00)	0/4 (0.00)

^1^ Chicken-related samples included tray liner, cloacal swab, carcass rinse, caecum, meat product.

^2^ Environmental samples included samples from hatchery (i.e., egg tray, egg incubator, tap water and egg shell), samples from broiler farm (i.e., boot swab, feeder, litter, water from nipple drinker, water from main pipeline, animal feed, footwear in the house and pest) and samples from slaughterhouse (i.e., transport crate, breast comforter, hanging shackle, eviscerating equipment, chilling water and packaging table).

^3^ NS, not sample.

^4^ n/a, not applicable.

^5^ No statistical analysis was conducted because all *Campylobacter* isolates from participating broiler flocks and slaughterhouses were identified as *C*. *jejuni*.

^a, b, c, d^ Prevalence of *Campylobacter* in the same production unit among 5 broiler production chains (i.e., A, B, C, D and E) were compared using chi-square test. Different superscripts indicate significant difference (*p*≤0.05).

During the rearing period, 0.80 to 48.75% of cloacal swab samples obtained from five broiler flocks were positive for *Campylobacter* (Table 2). *Campylobacter* prevalence in cloacal swab samples of farm C was lowest, whereas the highest prevalence of *Campylobacter* was observed in farm E. In contrast to breeder isolates, all isolates recovered from broiler flocks were identified as *C*. *jejuni*. At the first visit (7^th^ day), no *Campylobacter* was detected in any examined samples, with *Campylobacter* colonization first identified on the 14^th^ day in farms D and E. For large farms (A, B and C), *Campylobacter* could be isolated from chickens after 4 weeks of age. Within-flock prevalence varied among farms ranging from 3.33 to 93.33% (Table 3). Although the high prevalence could be found in cloacal swab samples, less than 7 percent of samples from the farm environment (e.g., boot swabs inside and outside the houses, darkling beetles, flies and drinking water) were contaminated with *Campylobacter*. Generally, *Campylobacter* positive environmental samples were found after chickens were colonized with these organisms.

**Table 3:** Within-flock prevalence and predominant genotypes of *Campylobacter* during the rearing period.

Production chain		Flock age (days)					
		14	21	28	31	35	38
A	Prevalence (percent)	0	0	0	70.00	36.67	86.67^a^
	Predominant sequence type (*flaA* SVR type)	n/a^b^	n/a	n/a	ST-574 (57)	ST-574 (57)	ST-45 (22)
B	Prevalence (percent)	0	0	0	90.00	86.67	90.00^a^
	Predominant sequence type (*flaA* SVR type)	n/a	n/a	n/a	ST-464 (54)	ST-464 (54)	ST-464 (54)
C	Prevalence (percent)	0	0	0	0	0	6.67^a^
	Predominant sequence type (*flaA* SVR type)	n/a	n/a	n/a	n/a	n/a	ST-2209 (629)
D	Prevalence (percent)	26.67	46.67	3.33	30.00^a^	n/a	n/a
	Predominant sequence type (*flaA* SVR type)	ST-1232 (783)	ST-1232 (783)	ST-1232 (783)	ST-1232 (783)	n/a	n/a
E	Prevalence (percent)	30.00	n/a	93.33	90.00^a^	n/a	n/a
	Predominant sequence type (*flaA* SVR type)	ST-5247 (287)	ST-5247 (287)	ST-5247 (287)	ST-1919 (253)	n/a	n/a

^a^ The last visit before the flock was sent to slaughterhouse.

^b^ n/a, not applicable.

In the slaughterhouse, a high prevalence of *Campylobacter* was found in chicken related samples (caecum, cloacal swab, meat product and carcass rinse) ranging from 37.88 to 90.00%. The prevalence of *Campylobacter* in chicken related samples of farms B and C was significantly lower than that of farms A, D and E (Table 2). Several types of slaughterhouse equipment and environmental samples (e.g., breast comforter, shackle, eviscerating equipment, chilling water and packaging table) were contaminated with *Campylobacter* with a range from 6.45 to 38.03%. The *Campylobacter* contamination rate in slaughterhouse equipment and environmental samples of farm E was significantly lower than that of farms A, B, C and D. Although *Campylobacter* were mostly recovered from the environment in the slaughterhouses after use, a few of the samples from the disinfected equipment (i.e., transport crate, eviscerating equipment and hanging shackle) were occasionally positive with *Campylobacter*. Similar to broiler flocks, *C*. *jejuni* was the predominant species found in slaughterhouses.

### Genetic characterization of *Campylobacter* isolated from poultry production chain

Amongst 311 *C*. *jejuni* isolates characterized by *flaA* SVR sequencing and 108 isolates further genotyped by multilocus sequence typing (MLST), 29 *flaA* SVR alleles and 17 sequence types were identified (Table 4). Fifteen sequence types were clustered into 10 clonal complexes, while 2 sequence types could not be grouped in any known clonal complex. Novel allelic sequences (asp 358, tkt 546 and tkt 553) and new sequence types (ST-6876, ST-6995 and ST-6996) were assigned. The most common clonal complex found in this study was CC-353 (e.g., ST-1075, ST-1232, ST-5213 and ST-5247), followed by CC-45 (e.g., ST-45 and ST-583). These clonal complexes were found to be distributed in every examined production chain, except for chain B.

**Table 4:** *Campylobacter* genotypes detected in each chicken meat production units.

Production chain	Production unit	Sample	Number of isolates examined	Genotype^a^	
				*flaA* SVR	MLST
A	Breeding farm	Cloacal swab	5	353, 506, 783, 1211, 1485	**1232**, 6876
	Broiler farm	Cloacal swab	36	18, 22, **57**, 312	45, 354, **574**
		Environment inside the target house^b^	4	18, 22, **57**	45, 354, **574**
		Environment outside the target house^c^	3	22, **57**	45, **574**
	Slaughterhouse	Cloacal swab and cecum	10	18, **22**, 57, 312	**45**, 354, 574
		Transport crate	3	**45**	**2409**
		Environmental sample^d^	5	18, **22**	**45**, 354
		Meat and carcass rinse	15	18, **22**, 57, 177	**45**, 354, 574, 583
B	Breeding farm	Cloacal swab	2	**54**	**464**
	Broiler farm	Cloacal swab	34	**54**, 18	**464**, 354
	Slaughterhouse	Cloacal swab and cecum	4	**54**	**464**
		Environmental sample	11	**54**, 783	**464**
		Meat and carcass rinse	10	**54**	**464**
C	Breeding farm	Cloacal swab	6	30, **34**, 54, 312	460, 574, **6996**
	Broiler farm	Environment inside the target house	1	22	45
		Cloacal swab	2	**629**	**2209**
	Slaughterhouse	Cloacal swab and cecum	4	**68, 629, 1340**	**2209**
		Transport crate	2	783	5213
		Environmental sample	2	783, 1340	5213
		Meat and carcass rinse	17	**18**, 68, 783, 1340	**354**, 2209
D	Breeding farm	Cloacal swab	1	677	2131
	Broiler farm	Cloacal swab	28	48, **783**	**1232**, 2131
		Environment inside the target house	4	**783**	**1232**
	Slaughterhouse	Cloacal swab and cecum	13	**783**	**1232, 5213**
		Environmental sample	5	22, **783**	1075
		Meat and carcass rinse	9	**783**	1232
E	Breeding farm	Cloacal swab	5	21, 54, 45, 402, 48	2131
	Broiler farm	Cloacal swab	52	18, 45, 57, 253, 255, **287**, 854, 1527	1919, **5247**
		Environment inside the target house	2	287, 1239	5247
		Environment outside the target house	3	255, 287, 1397	6995
	Slaughterhouse	Cloacal swab and cecum	4	253, **783**,1527	n/a^e^
		Environmental sample	3	45, 253, 652	n/a
		Meat and carcass rinse	6	45, **287**, 312, 652	**5247**

^a^ Three-hundred and eleven isolates were typed by *flaA* SVR and 108 isolates were further characterized by MLST. Bold letter stands for predominant strain.

^b^ Environment inside the target house: boot swab inside the target house, water from nipple drinkers, darkling beetles and flies.

^c^ Environment outside the target house: boot swab from path-leading to target house, boot swab from area around the house and boot swab inside the adjacent house.

^d^ Environmental sample: eviscerating equipment, shackle, chilling water and packaging table.

^e^ n/a, not applicable.

**Fig 2:**
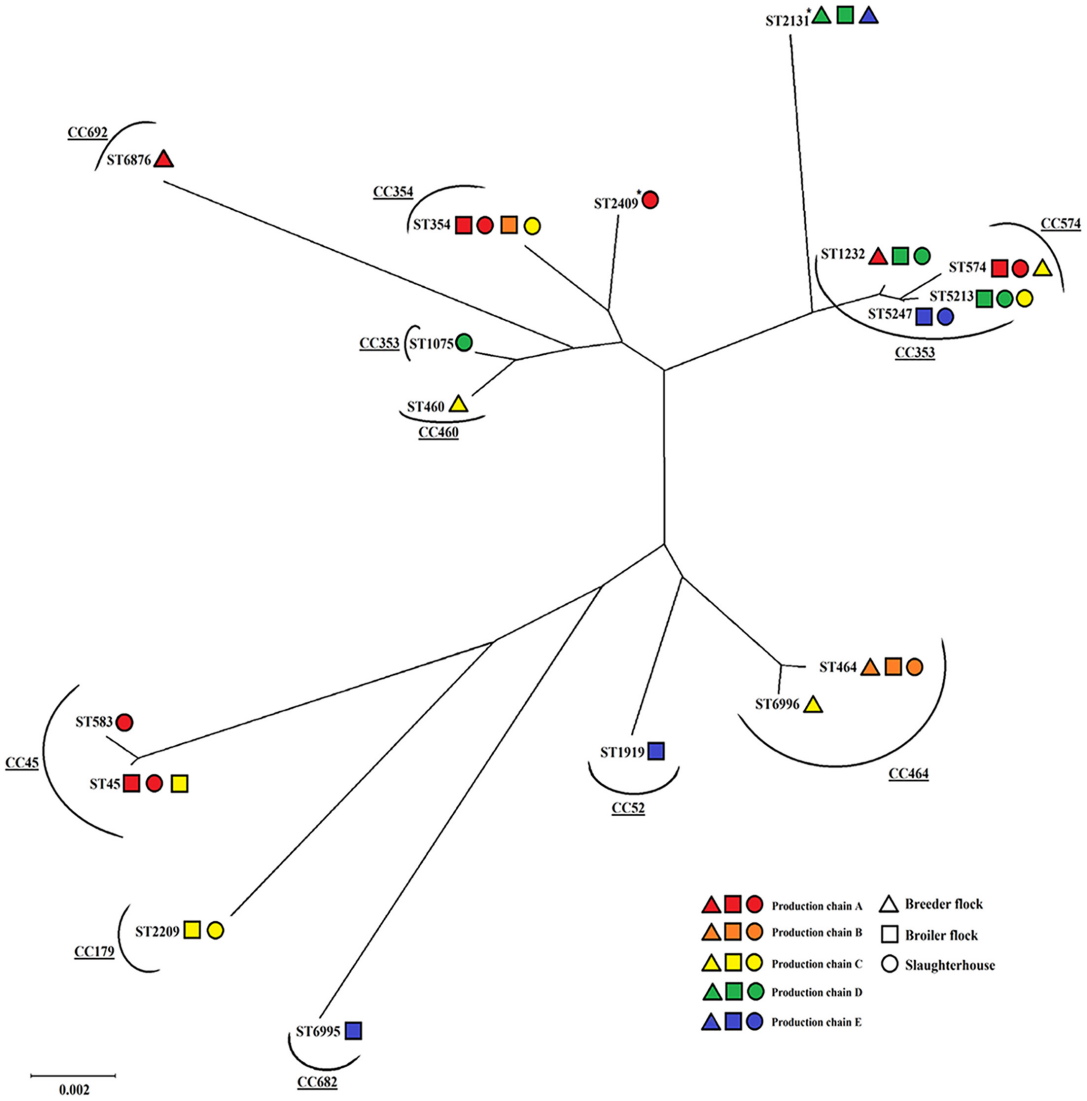
Phylogenetic relationship of *Campylobacter jejuni* from various sources of broiler production processes. Distribution of sequence types in each production chain (i.e., A, B, C, D and E) and production unit (breeder farm, broiler farm and slaughterhouse) was represented by different shading pattern and geometric shape, respectively. Asterisk (*) defined as unassigned clonal complexes.

In chains A, C, D and E, most of sequence types and *flaA* SVR genotypes of *C*. *jejuni* isolated from breeders and their respective progenies were distantly related (Fig 2). In contrast, genetic similarity between *C*. *jejuni* isolated from breeders and broilers was observed in chain B. A single dominant genotype (ST-464 or *flaA* SVR allele 54) was identified throughout the chicken meat production chain B, even though a few strains i.e., ST-354 (or *flaA* SVR allele 18) and *flaA* SVR allele 783 were occasionally present. However, for the other production chains, multiple genotypes of *C*. *jejuni* were identified (Table 4). Substitution of the initial predominant genotype in broiler flock A was demonstrated, where the predominant strain changed from ST-574 (or *flaA* SVR allele 57) to ST-45 (or *flaA* SVR allele 22) during the rearing period (Table 3). This ST-45 strain also remained the predominant sequence type in the slaughterhouse. For the late colonized flock (flock C), a single strain (ST-2209 or *flaA* SVR allele 629) was identified. Although this sequence type was predominantly found in the chicken intestinal tract until slaughter, it was dominated by another sequence type (ST-354 or *flaA* SVR allele 18) on chicken carcasses (Table 4). Genetic diversity of *C*. *jejuni* was more frequently noticed at the end of the rearing period. This finding was obvious in flock E where multiple *flaA* SVR genotypes (i.e., 18, 45, 253, 255, 287, 854 and 1527) were detected, particularly at the day before the birds were sent to slaughterhouse (Table 4). In general, most *C*. *jejuni* contaminating the slaughterhouse environment, equipment, carcass rinses and meat products were genetically similar to those found in broiler flocks and caeca.

To reveal the source of *Campylobacter* in broiler farms, genetic comparison between *C*. *jejuni* isolated from the broiler house surroundings before chick placement and *C*. *jejuni* isolated from broiler flocks was conducted. In this study, no *Campylobacter* was recovered from the farm environment before chick placement, while only one isolate was obtained from environmental samples collected before *Campylobacter* detection in broilers. However, this isolate which was recovered from water from a nipple drinker of flock C (ST-45) was genetically different from those that colonized in the broiler flock (ST-2209). Generally, *Campylobacter* were recovered from the house environment after flocks became positive and the predominant sequence types identified in both house environment and broiler flocks were quite similar. For instance, predominant strains of the birds in flocks A, D and E (i.e., ST-574, ST-1232 and ST-5247, respectively) were found to be the main sequence types in environmental samples such as boot swabs, water from nipple drinkers, flies and darkling beetles. The above findings indicated that the majority of *C*. *jejuni* present in the farm environment originated from broilers.

## Discussion

Over the last decade, the prevalence of *Campylobacter* in the poultry production chain has been widely investigated in many countries. Although strategies for reducing the incidence of this pathogen in poultry and poultry products are being studied and applied, the prevalence of *Campylobacter* is still high [5]. To improve the efficiency of *Campylobacter* interventions, the epidemiology and population biology of these bacteria in poultry need to be elucidated. The present study demonstrated the distribution and population structure of *Campylobacter* in Thai poultry production processes.

In this study, all breeder flocks were colonized with *Campylobacter*, while the organism was not recovered from hatchery samples or tray liners of day-old-chicks. Differences in *Campylobacter* genotypes identified in breeders and their following production units indicate that vertical transmission might not be the major route of *Campylobacter* transmission in Thai broiler production chain.

In the present study, a high proportion of *C*. *coli* in breeder flocks was reported, while no *C*. *coli* was recovered from broiler and slaughterhouse samples. This finding was similar to the study of O’Mahony et al. (2011) who described *C*. *coli* as the predominant species in breeders. In general, *C*. *jejuni* was reported as the predominant *Campylobacter* species in the poultry population, but a higher proportion of *C*. *coli* could be found in certain types of poultry productions such as free-ranged chickens, laying hens or chicken breeders [30–32]. An earlier study suggested that the increase in the *C*. *coli* proportion in chickens was usually associated with flock age [33]. In addition, the use of disinfectants and antibiotics on farms could select for certain bacterial populations [34]. In this study, the use of antimicrobial agents, including lincosamides, macrolides and tetracyclines, were reported in participating breeder flocks (Personnel communication). Macrolides (e.g., tylosin) are usually applied for prophylaxis and treatment of respiratory diseases, particularly mycoplasmosis in poultry [35]. Since *C*. *coli* was usually found to be more resistant to macrolides than *C*. *jejuni* [36], it is possible that the use of macrolides in breeder flocks may select for *C*. *coli*. Until now, there was no solid evidence to confirm this hypothesis. Therefore, it might be worth determining the effect of bird age and macrolide usage on *C*. *coli* proportions in breeder flocks in future studies.

The presence of multiple strains of *Campylobacter* was identified in each broiler flock, particularly at the end of the rearing period. Additional strains were intermittently recovered from the flocks along the rearing period; these could indicate breaches of biosecurity on the farms allowing ingress of *Campylobacter* into the broiler house. Interestingly, most of those new strains were distantly related to the preexisting strains (Fig 2). In the past, several sources e.g., domestic and wild animals, contaminated water, farm staff and house equipment were identified as risk factors associated with *Campylobacter* colonization in broilers [12]. However, the evidence of potential sources of *Campylobacter* is unclear in this study. Improvement in personnel hygiene practices and biosecurity on the poultry farm should be the primary strategy to prevent *Campylobacter* introduction into broiler flocks.

Implementation of strict biosecurity practices was considered as the effective method to prevent or postpone *Campylobacter* colonization time in broiler flocks during the rearing period [37]. From the studies in Norway and Denmark, improvement of biosecurity was mentioned as the significant protective factor for *Campylobacter* colonization in poultry farms [38, 39]. In broiler farms B and C, which were located adjacent to each other, the predominant sequence types present in these farms (ST-464 and ST-2209, respectively) were unrelated (Fig 2). This finding indicated that proper farm management and farm biosecurity might be the effective way for *Campylobacter* prevention and control in broiler flocks.

From previous investigations, broiler flocks reared on larger farms were more likely to be colonized with *Campylobacter* than those reared on small farms [40]. In contrast, early colonization (14^th^ day) observed in the present study was found in small-scale farms (i.e., farms D and E). Meanwhile, *Campylobacter* were firstly detected in the late rearing period (31^st^ to 38^th^ day) of larger farms (i.e., farms A, B and C). According to farm data, large-scale farms in this study were operated with strict biosecurity and good management practices, while lower level of farm biosecurity was described in small-scale farms. Differences in farm management and biosecurity practices might be one of the explanations for this finding.

Meat products from *Campylobacter-*positive broiler flocks were more likely to be contaminated with this organism than the products from *Campylobacter*-free flocks [41]. Increasing numbers of *Campylobacter* on carcasses was commonly reported after plucking and eviscerating procedures [42]. In the present study, genetic relatedness between *Campylobacter* isolated from the intestinal tract of broilers and samples collected from slaughterhouses e.g., eviscerating equipment, shackles, carcass rinses and meat products, was revealed. The existence of *Campylobacter* after disinfection is of concern. To minimize the spreading of *Campylobacter* on poultry carcasses, the prevention of intestinal content leakage as well as effective cleaning and disinfection of the slaughterhouse environment during the slaughtering process should be emphasized. Management interventions e.g., logistic slaughter were also suggested as supporting preventive methods [42].

The main clonal complexes identified in this study were ST-45, ST-353, ST-354 and ST-574 complex. ST-45 complex is known as one of the most common clonal complexes identified in human cases, various types of animal hosts and environmental samples [43]. There is evidence indicating that members of the ST-45 complex were environmentally adapted strains, which can survive under unfavorable conditions better than other strains [44, 45]. Similar to the ST-45 complex, the ST-353 has also been described as one of the common clonal complexes recovered from human cases and poultry [46, 47]. In the present study, at least one isolate from each production chain, except for chain B, belonged to the ST-353 complex. Although the ST-354 and ST-574 complexes are not common at the global level, they were commonly found in this study. According to the MLST database, ST-354 and ST-574 were reported as the predominant strains found in human and poultry samples of Thailand. Interestingly, our study could not detect any ST-21 complex which was extensively known as the most common clonal complex identified in wide-ranging sources and associated with human infection worldwide. However, this clonal complex was not predominantly detected in Thailand. In addition, according to the MLST database (http://pubmlst.org/campylobacter/), most of the clonal complexes identified in the present study were similar to clonal complexes previously reported in human cases in Thailand. This finding emphasizes the importance of poultry as one of the significant sources of *Campylobacter* infection in humans.

Although our study provides significant information on the distribution and genetic profiles of *Campylobacter* in commercial broiler production in Thailand, there are some points which might be regarded as weaknesses of this study. The number of samples collected was lower than the sample size determined by statistical calculation. At 90% confidence level, the calculated sample size of cloacal swab samples that should be collected from broiler farms A, B, C, D and E was 244, 247, 248, 246 and 244 samples, respectively. However, the actual number of cloacal swab samples collected from these farms was 210, 210, 240, 150 and 150 samples for broiler farms A, B, C, D and E, respectively. Since chickens in farms D and E were sent to slaughterhouses faster than those in farms A, B and C and since sample collection from farms D and E was less frequent than that from farms A, B and C (due to the limited permission received from farm owners), the number of samples collected from farms D and E was lower than that from the other farms. Although these limitations might have some effects on the results and conclusion of the present study, their impact should not be that much especially if the ratio between production capacity and sample size was considered. Several factors including budget and cooperation of farm owners can influence sample collection plan. However, in the present study, we tried our best to minimize the problems caused by those limitations.

Our findings reveal that *Campylobacter* were distributed throughout the Thai broiler production process. Flock colonization and carcass contamination with various genotypes of *Campylobacter* reflect the presence of several sources of *Campylobacter* during the poultry production process. To minimize *Campylobacter* contamination in chicken, interventions should be conducted both at the broiler farm and in the slaughterhouse. This study suggests that standard hygienic practices and biosecurity seem to be the most practical strategies for prevention and control of *Campylobacter* during broiler production process.

## Supporting Information

S1 Table Sample collection procedures used in this study. (DOCX)
