# Supplementary material for: Distribution and Genetic Profiles of Campylobacter in Commercial Broiler Production from Breeder to Slaughter in Thailand
Source: PLoS One. 2016 Feb 17;11(2):e0149585. doi: 10.1371/journal.pone.0149585 (PMC4757449; doi:10.1371/journal.pone.0149585)
Supplement: S1 Table — (DOCX) [file pone.0149585.s001.docx]

S1 Table Sample collection procedures used in this study

| **Production unit** | **Type of sample** | **Sample collection procedure** |
| --- | --- | --- |
| Breeder flock | Cloacal swab | Moisten cotton swab was inserted into cloaca. The cotton swab was gently moved in a circular motion for two to three times. Then, the swab was put in Cary-Blair transport medium. |
| Hatchery | Egg incubator | Three inner sides of the egg incubator’s wall were swabbed by moisten gauze. After each inner side was wiped thoroughly for approximately 900 cm^2^, gauze was kept in a sterile plastic bag. |
|  | Egg shell | Approximately 100 grams of egg shell were collected from each egg tray. Ten egg trays were sampled per flock. |
|  | Egg tray | Four corner areas of each egg tray were sampled using a sterile cotton swab. Each area was swabbed for approximately 100 cm^2^. Then, the swab was put in Cary-Blair transport medium. In total, 10 egg trays were sampled per flock. |
|  | Tap water | At least 1,000 ml of tap water circulated in the hatchery system were collected in a sterile plastic container. |
| Broiler flock | Cloacal swab | As mentioned in breeder flock section. |
|  | Animal feed | Approximately 100 grams of animal feed were collected from feed silo. |
|  | Boot swab | Moisten shoe covers (boot swabs) were put on the footwear. Then, sample collection was conducted by walking inside the house, around the house or on the path leading to the house. Shoe covers were carefully taken off and kept in a sterile plastic bag. |
|  | Feeder | Both pan feeder and trough feeder were sampled using gauze swab. The whole area inside pan feeder was swabbed using a single gauze. For trough feeder, inner surface of the feeder was swabbed lengthwise. In total, 12 feeders (i.e., 6 trough feeders and 6 pan feeders) were sampled for each investigation. |

| **Production unit** | **Type of sample** | **Sample collection procedure** |
| --- | --- | --- |
| Broiler flock (Cont.) | Litter | Litter was collected from 3 areas in the broiler house: anterior part of the house, middle part of the house and posterior part of the house. At least 100 grams of litter were collected from each area. |
|  | Nipple drinker | Water from nipple drinkers was collected from 3 areas along the water pipes in the house i.e., the anterior part of the house, middle part of the house and posterior part of the house.Approximately 100 ml of water from 5 nipple drinkers were collected from each area. To collect water sample, sterile cotton swab was used to press on the tip of nipple drinker to fill water into a sterile container. |
|  | Tray liner | Tray liners were collected from transport crates after chicks were brought to the farm. Tray liners from 10 transport crates were sampled for each investigation. |
|  | Water inlet | Water from the main water inlet to broiler house was collected for at least 1,000 ml in a sterile plastic container. |
| Slaughterhouse | Cloacal swab | As mentioned in breeder flock section. |
|  | Cecum | Ten intact ceca were collected at the evisceration process. Each cecum was packed separately in a sterile plastic bag. Samples were kept on ice during transport. |
|  | Chilling water | Water from the chilling tank was collected for at least 1,000 ml in a sterile plastic container. |
|  | Shackle | Five shackles were swabbed using a sterile cotton swab. Then, the swab was put in Cary-Blair transport medium. This process was repeated for 5 times. Thus, a total of 25 shackles were sampled for each flock. |
|  | Slaughterhouse equipment | The surface of slaughterhouse equipment was sampled using a sterile cotton swab. Three types of slaughterhouse equipment (i.e., knife, eviscerating fork and vent gun) were sampled. Five samples were collected from each equipment type. |

| **Production unit** | **Type of sample** | **Sample collection procedure** |
| --- | --- | --- |
| Slaughterhouse (Cont.) | Tap water | At least 1,000 ml of tap water circulated in the slaughterhouse system were collected in a sterile plastic container. |
|  | Transport crate | Four corner areas of each transport crate were sampled using a sterile gauze. After the inner surface of the transport crate was swabbed, gauze was kept in a sterile plastic bag. Five transport crates were sampled per flock. |
